# A cretaceous frog with eggs from northwestern China provides fossil evidence for sexual maturity preceding skeletal maturity in anurans

**DOI:** 10.1098/rspb.2023.2320

**Published:** 2024-02-07

**Authors:** Baoxia Du, Jing Zhang, Raúl Orencio Gómez, Liping Dong, Mingzhen Zhang, Xiangtong Lei, Aijing Li, Shuang Dai

**Affiliations:** ^1^ School of Earth Sciences and Key Laboratory of Mineral Resources in Western China (Gansu Province), Lanzhou University, Lanzhou 730000, People's Republic of China; ^2^ Laboratorio de Morfología Evolutiva y Paleobiología de Vertebrados, Departamento de Biodiversidad y Biología Experimental, Facultad de Ciencias Exactas y Naturales, Universidad de Buenos Aires, Buenos Aires C1428EGA, Argentina; ^3^ Key Laboratory of Vertebrate Evolution and Human Origin of Chinese Academy of Sciences, Institute of Vertebrate Paleontology and Paleoanthropology, Chinese Academy of Sciences, Beijing 100044, People's Republic of China; ^4^ Northwest Institute of Eco-Environment and Resources, Chinese Academy of Sciences/Key Laboratory of Petroleum Resources, Lanzhou, Gansu Province 730000, People's Republic of China; ^5^ Yunnan Key Laboratory for Palaeobiology, Institute of Palaeontology, Yunnan University, Kunming, Yunnan Province 650000, People's Republic of China

**Keywords:** Anura, Early Cretaceous, northwest China, *Gansubatrachus*, sexual maturation

## Abstract

Mesozoic fossils of frogs are rare in the palaeontological record, particularly those exhibiting soft tissues that offer limited insights into early life-history characteristics. Here we report on a skeletally immature frog from the Lower Cretaceous of northwest China, with egg masses in the body and eggs in the oviduct, indicative of a gravid female. CT reconstruction of the specimen allows referral to *Gansubatrachus qilianensis* and we assign it as a paratype complementing the diagnosis of the type species*.* The new fossil, which might represent a younger individual than the holotype of *Gansubatrachus*, shows that sexual maturation occurred before full adulthood in this frog and provides evidence of death linked to mating behaviour. We also discuss other potential sources of variation and life-history traits of *Gansubatrachus*. The new finding represents the oldest Early Cretaceous frog preserving *in situ* eggs and provides a glimpse into ancient anuran development during Mesozoic times.

## 1. Introduction

Anurans (frogs) are unique among living land vertebrates, being extremely diverse in their development, reproductive modes and other life-history traits [1,2], whereas they have been conserved in their skeletal body plan since Jurassic times [3,4]. Most anurans have a biphasic life cycle characterized by different larval (tadpole) and adult (frog) body forms [5,6], but some frogs have evolved direct development, lacking a free-living larva [7]. In all cases, though, they develop from soft, ‘jelly’-coated eggs [8], and exhibit a high diversity in the size and number of eggs produced by female frogs [1,9]. In contrast with the extraordinary current diversity of anurans (more than 7600 species worldwide [10]), their fossil record is relatively sparse, yet fossils of both tadpoles and adults have been described [11–13]. Most of these fossils typically preserve skeletal data only and, in a few cases, part of the body outline, being extremely rare in the preservation of soft body parts, including eggs. Fossil eggs of anurans have been reported from a few localities with ages ranging from the Mid-Cretaceous to the Pliocene [13,14]. These include a single isolated egg preserved in Cretaceous amber [14], eggs associated with Early Oligocene adult palaeobatrachids of Bechlejovice, Czech Republic [15] (although later interpreted as bubble-like traces caused by gas vesicles [16]), Middle Eocene adult pelobatids of Messel, Germany [17], a single adult ranid from the Pliocene of Willershausen, Germany [18,19], and an isolated egg containing an emerging tadpole in Miocene amber from the Dominican Republic [20].

Reproductive characteristics are critical components of life history [21–23], with size at sexual maturity and sexual dimorphism being important traits for understanding the population dynamics of species [23]. The stage at which sexual maturity occurs greatly influences fitness and is a critical transition in the life history of organisms [24–26]. In extant species, this stage is most often determined by gonadal characteristics or external secondary sexual traits [27], although skeletochronology has also been applied to determine the relative age of anurans and other vertebrates [28–30]. However, in the case of fossils most of these approaches are typically hindered by incomplete preservation of body parts. Skeletochronology has been used to determine the age with relative success in a few studies [31], although it does not allow for direct inference of sexual characteristics. In exceptional cases, sexual maturity of extinct vertebrates such as dinosaurs has been established directly from closely associated eggs [32,33], but these findings are very rare in the fossil records.

Here we report on such a rare fossil, a Lower Cretaceous gravid female frog from the Jiuquan Basin of northwest China. It provides complementary data of the skeleton and life history of the recently described *Gansubatrachus qilianensis* [34], which inhabited northwestern China more than 100 Myr ago.

## 2. Material and methods

**Figure 1.**
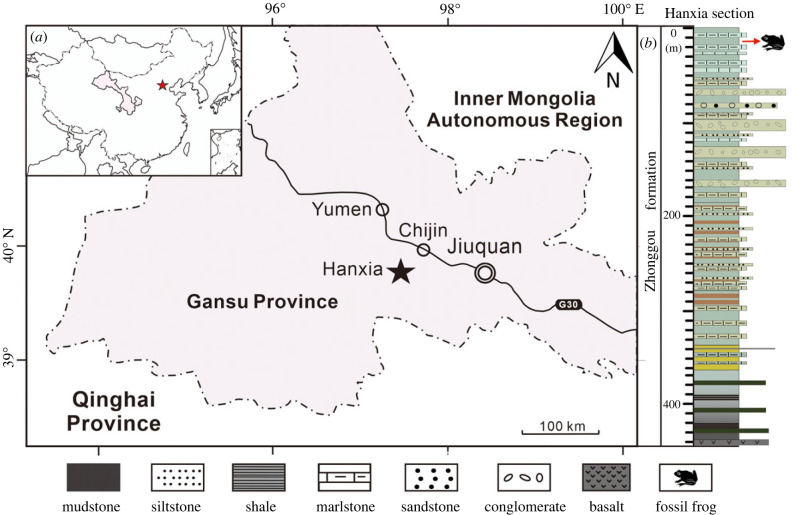
Holotype and paratype locality (star) for *Gansubatrachus qiliansis* (*a*) and stratigraphic details for the Zhonggou Formation (*b*) [34].

The specimen described herein is preserved on part and counterpart slabs of JQ-HX-QW-02A,B and belongs to the collection of the Paleontological Laboratory of the School of Earth Sciences, Lanzhou University, in Lanzhou, China. It was collected from the uppermost Zhonggou Formation of the Hanxia outcrop, about 25 km southwest of Chijin Town, Yumen City, Gansu Province, China (figure 1*a*). We performed high-resolution X-ray computed tomography (micro-CT) on the part JQ-HX-QW-02A, but not the counterpart JQ-HX-QW-02B at Yunnan University. The obtained micro-CT data consists of 2399 slices taken along the long axis of the specimen; the pixel size of each slice is 43.96 µm, with an interstice spacing of 43.96 µm. Then we used Avizo software (FEI Visualization Sciences Group, Hillsboro, Oregon, USA) to digitally reconstruct the fossil model from raw micro-CT data through the function of 3D volume rendering.

To explore the nature of the fossilized eggs, elemental maps of specific areas of the fossil were produced using energy dispersive X-ray spectrometer (EDS) at the Yunnan Key Laboratory for Palaeobiology. We designated JQ-HX-QW-02A,B as the paratype of *Gansubatrachus qilianensis,* which was previously known only from its holotype (JQ-HX-QW-01), based on the shared presence of several skeletal and one soft-body features [34]. Data from the new paratype were combined with data of the holotype in the phylogenetic analyses to test the relationships of *Gansubatrachus qilianensis.* The analyses used the same approach as Zhang *et al*. [34] and character scorings of *Gansubatrachus qilianensis* are provided in the electronic supplementary material. General osteological terminology follows Maglia *et al*. [35] and Turazzini & Gómez [36]. Carpal terms follow Fabrezi [37] and pelvic terms follow Gómez and Turazzini [38]. Original micro-CT data are available for download on Morphosource (http://www.morphosource.org; ID: 000531113).

## 3. Locality and horizon

The paratype (JQ-HX-QW-02A, B) was found at the same horizon as the holotype (JQ-HX-QW-01), both in the third layer of the uppermost Zhonggou Formation of the Hanxia outcrop, and the fossils are preserved in the horizontally stratified extremely well-developed grey-green mudstones (figure 1*b*), as defined by Du's description [39]. The uppermost part of the Zhonggou Formation is typical of lacustrine deposits, and is rich in well-preserved plants, fishes, insects and conchostracan fossils [40]. The age of the Zhonggou Formation has been dated as Late Aptian–Early Albian based on single zircon U-Pb isotope analysis, macrofossil and palynological assemblages [40–43].

## 4. Results

Anura Fischer, 1813

*Gansubatrachus* Zhang *et al*. [34]

*Gansubatrachus qilianensis* Zhang *et al*. [34].

### (a) Holotype

JQ-HX-QW-01, an incomplete skeleton of an adult frog preserved on a single mudstone slab (figure 5*a*_1_, *a*_2_).

### (b) Paratype

**Figure 2.**
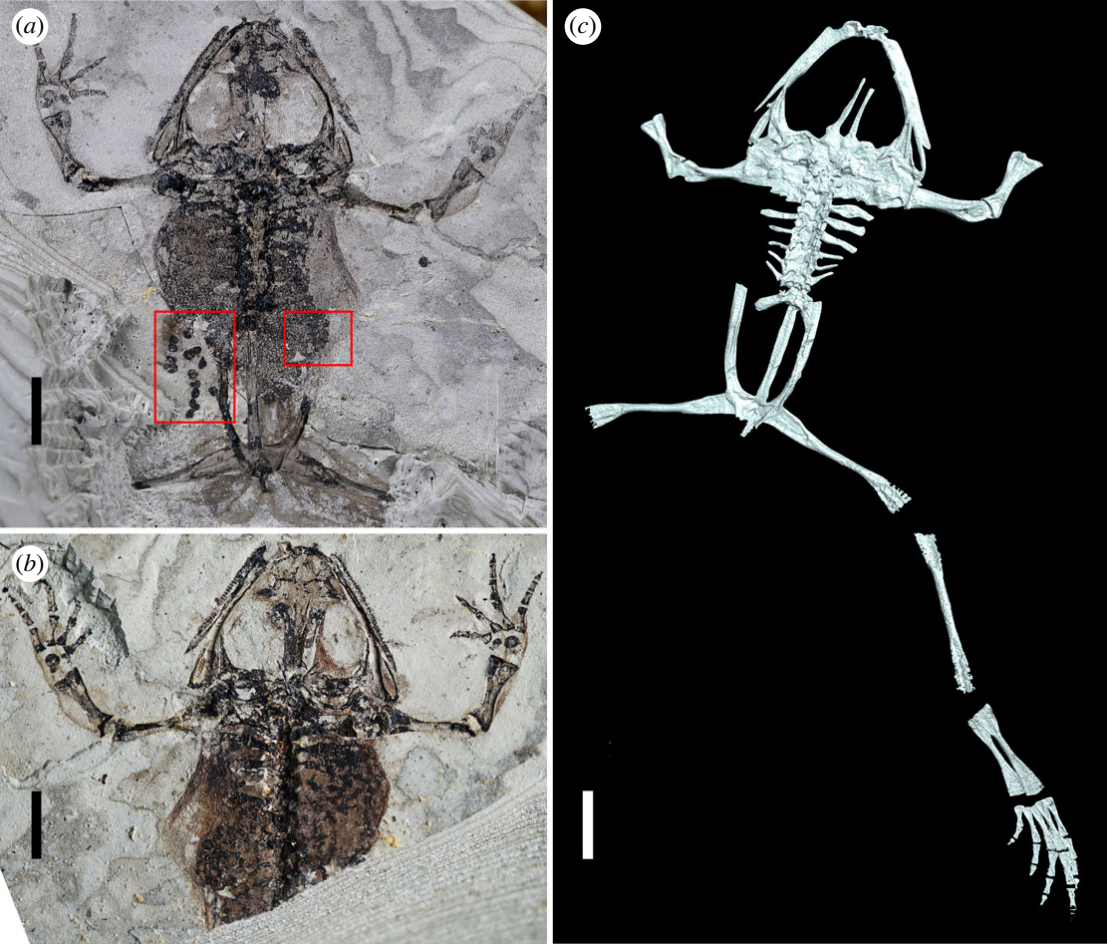
Paratype of *Gansubatrachus qilianensis.* (*a,b*) Photography of part and counterpart (JQ-HX-QW-02A and JQ-HX-QW-02B), showing the skeleton and soft-body parts, with the red box in dorsal view boxes indicating eggs preserved in the body cavity; (*c*) CT reconstruction of dorsal view, showing the intact right hind limb covered in rock. The parasphenoid bone was excluded from reconstruction because it was obscured by the frontalparietal. Scale bar: 5 mm.

**Figure 3.**
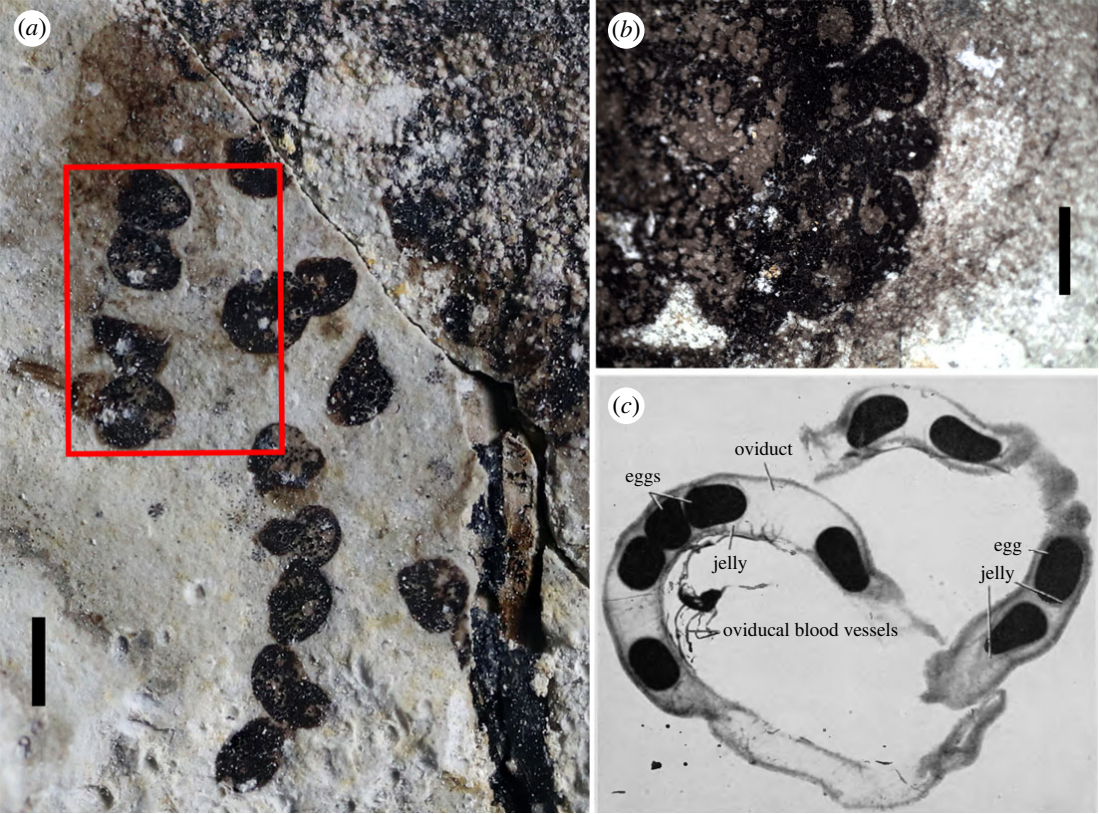
Anuran eggs within body cavities of the fossil frog *Gansubatrachus qilianensis* (paratype JQ-HX-QW-02) and extant ranid frog. (*a*) *G. qilianensis*, enlarged view of left red box in figure 2*a*, showing fossil eggs arranged in columns and likely enclosed within oviduct; (*b*) *G. qilianensis*, enlarged view of right red box in figure 2*a* showing cluster of eggs, possibly within ovary; (*c*) passage of eggs through the paired oviducts in the extant frog *Rana pipiens* (Image revised from the Biodiversity Heritage Library. Contributed by MBLWHOI Libraries. (www.biodiversitylibrary.org) [64]). Scale bars in *a* and *b*: 1 mm.

JQ-HX-QW-02A, B, part and counterpart of an incomplete adult skeleton with eggs, preserved on mudstone slabs (figures 2*a,b*, 3*a,b* and 5*b*_1_, *b*_2_).

Revised diagnosis (modified from Zhang *et al*. [34]): *Gansubatrachus qilianensis* is a small frog that differs from all other anuran genera in the following unique combination of features: small body size (snout–vent length [SVL] 30–40 mm); skull wider than long; robust and non-bifurcated alary process of premaxilla; quadratojugal present; large fontanelle between paired frontoparietals; paired nasals meeting in the front half along the midline; vomer with a dentigerous portion bearing 6–10 teeth arranged in a single row; palatine absent; ‘V’-shaped parahyoid; columella present; paired sphenethmoids; atlas with type II cotyles; eight presacral vertebrae; separate ribs present on presacrals II–IV; unexpanded and posteriorly directed sacral diapophyses; arciferal pectoral girdle; short and stocky scapula; straight leading edge of the scapula, the anterior of which is overlapped medially with the clavicle; imbricate neural arches; bicondylar sacro-urostylar articulation; ilium with low dorsal crest; relatively long hind limbs.

### (c) Remarks

Specimen JQ-HX-QW-02 preserves the skeleton of a small-sized frog (snout–vent length = 35 mm) with the soft tissue outline of the body clearly visible, being about the same size of the holotype [34]. The skeleton matches the description of *Gansubatrachus qilianensis* [34], a frog previously recorded from the same outcrop in Gansu Province, in the presence of a robust and non-bifurcated alary process on the premaxilla, a ‘V’-shaped parahyoid, a paired sphenethmoid, eight presacral vertebrae, three pairs of free ribs, and unexpanded sacral diapophyses. The CT-scanning (figure 2*c*) was used to reconstruct the hind limb bones and supplemented some features missing in the holotype, such as the imbricate neural arches, bicondylar sacro-urostylar articulation, ilium with low dorsal crest and relatively long hind limbs. New phylogenetic analysis based on the evidence from both the holotype and the paratype, agrees with previous results [34]*,* finding it as part of Lalagobatrachia, probably as a basal member of this clade (electronic supplementary material).

## 5. Brief description of paratype

The frontoparietals are relatively well preserved in outline, and although slightly displaced overall, they exhibit clear bilateral symmetry with posterior closure through midline contact between the frontoparietals, while also featuring a prominent frontal–parietal fontanelle. The squamosal is well preserved, having a T-shaped configuration. The anterior end of the maxilla has a small anterodorsal process and a rounded anteroventral process that articulates with the premaxilla. The sphenethmoid is preserved as a pair of ossifications on the anterolateral part of the braincase. The T-shaped parasphenoid is poorly preserved but might be slightly different from the holotype in its cultriform process, the anterior portion of which appears to narrow abruptly into a needle-like tip.

The axial skeleton includes eight presacrals, a single sacral vertebra and a urostyle. The micro-CT reconstruction shows imbricate neural arches, but the structure of the centra is still difficult to assess. The sacral vertebra has two posterior condyles for a bicondylar articulation with the urostyle. The length of the urostyle is slightly shorter than the length of the presacral region. The urostyle is dorsally smooth and lacks a dorsal crest, but has a groove running dorsally along its long axis.

The curved clavicle and oblique position to the coracoid indicate an arciferal pectoral girdle. The pelvis is well preserved, although it has been slightly distorted and displaced from its articulation with the sacral vertebra. The ilium has a cylindrical shaft that has a low dorsal crest. A low but distinct dorsal prominence projects dorsally at the base of the ilial shaft. The ischium has a dorsal expansion above the acetabulum and a posterior projection in dorsal view.

The epiphyses of long bones are not preserved, indicating that they were cartilaginous. The forelimb elements of the two specimens differ only in the ossification status of the carpal bones, in which the carpal bones in the paratype (JQ-HX-QW-02) are not completely ossified, and only the distal ulnar and radial elements are preserved. This also shows the paratype specimen was at a young post-metamorphic stage of frog development. The paratype is supplemented with hindlimb features, wherein the intact right hindlimb is encased within the rock matrix, and its morphology is visualized by CT reconstruction (figure 2*c*). The femur is weakly sigmoid, having a slender shaft and slightly expanded proximal and distal ends. The tibiofibula is slightly longer than the femur. The tibiale and fibulare are unfused, but contact one another at both ends, leaving a narrow intertarsal fenestra between the two bones. Metatarsal (Mt) IV is the longest (10 mm), followed by Mt III (8 mm). Mt II and V are roughly equal in length (7.5 mm), whereas Mt I is the shortest, being only 4 mm long. The phalangeal formula of the pes may be 2-2-3-4(?)-3, a typical pattern for most frogs.

## 6. Soft body parts

JQ-HX-QW-02 preserves the body outline and carbonaceous soft tissue, including part of the viscera on both slabs (figures 2*a, b* and 3*a,b*). The outline of the body reveals that, as in the holotype of *Gansubatrachus qilianensis* [34], the manus was partially webbed (figure 2*a,b*). The fossilized body exhibits a well-preserved and tightly constrained upper cavity, while decay and disruption are evident in the lower half of the body cavity, as it appears to have lost its contour (figure 2*a*). On the left side of the fossil specimen, preservation is somewhat ambiguous, potentially due to compressional forces, making it challenging to discern individual components. A string of irregular eggs located near the ilium on the left part of JQ-HX-QW-02A was identified. Fourteen slightly deformed eggs, which are of similar size, approximately 0.8 mm in diameter, were observed with some residual colloid near the top (figure 3*a*). By contrast, the right side of the body cavity exhibits distinct demarcation between the skin and its contents. An intriguing observation is the presence of mass in the lower part of the body cavity, consisting of uniformly sized, also approximately 0.8 mm in diameter, black spherical corpuscles (figure 3*b*). These structures bear resemblance to mature ovarian follicles in terms of their position and morphological characteristics. The presence of a developing egg within each ovarian follicle, however, is challenging to observe on the fossil specimen.

**Figure 4.**
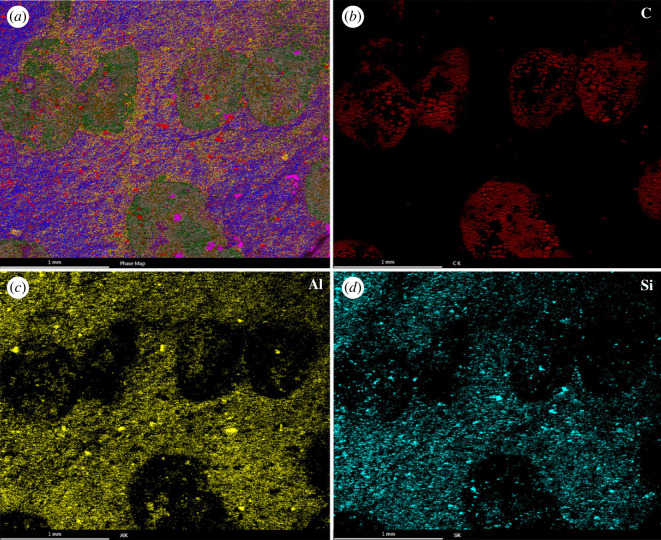
Energy dispersive X-ray spectroscopic (EDS) elemental maps illustrating preservation of eggs ascarbonized fossil. (*a*) EDS analysis of the field of view shown rotated 90° clockwise by the red box in figure 3*a*; (*b–d*) C, Al and Si maps are the same as the field of view of figure 4*a*.

Local areas of the soft tissues (i.e. carbonized eggs) in JQ-HX-QW-02A (figure 3*a*) were chemically analysed. The EDS measurements performed provide an overall distribution of at least ten recognizable elements in the specimen (aluminium, calcium, carbon, iron, magnesium, nitrogen, oxygen, phosphorus, silicon, sodium and sulfur), of which we only showed the distributions maps of the major three elements (carbon, aluminium and silicon) in the fossil and sediment (figure 4), with maps of the other elements available in the electronic supplementary material. Among these, the soft tissues area of the fossil is itself carbonized, and the sediment is maximally rich in aluminium and silicon.

## 7. Discussion

### (a) Developmental stage of the specimen JQ-HX-QW-02

The age or stage of post-metamorphic anurans cannot be inferred directly from their body length, because body size is strongly affected by environmental conditions and sexual dimorphism [44,45]. Sexual dimorphism in the development of anurans is complex, and it is not readily apparent in young individuals [46]. Despite the incomplete preservation of the holotype, the size and proportions of the forelimb elements in both the holotype and paratype could be compared (holotype, humerus: 6.2 mm, radio-ulna: 4.87 mm; paratype, humerus: 5.92 mm, radio-ulna: 4.80 mm), showing that the holotype and paratype are about the same size. This is consistent with the total length of the paratype (35 mm) falling between the previously estimated total length for *Gansubatrachus qilianensis* (30–40 mm) [34]. Instead, staging post-metamorphic anurans, both extant and extinct, relies on a fairly consistent set of developmental changes in their skeleton [47,48], although some variation exists in the relative sequence of cranial versus postcranial ossification in some groups [49]. The relative age of an individual within a species can therefore be estimated based on the degree of ossification of some skeletal parts (such as that of the carpus). According to studies on the ossification sequences of extant frogs [11,48–52], the frontoparietals are among the earliest bones to undergo ossification in the skull, yet it remains thin and fragile during early post-metamorphic stages [11,53,54].

**Figure 5.**
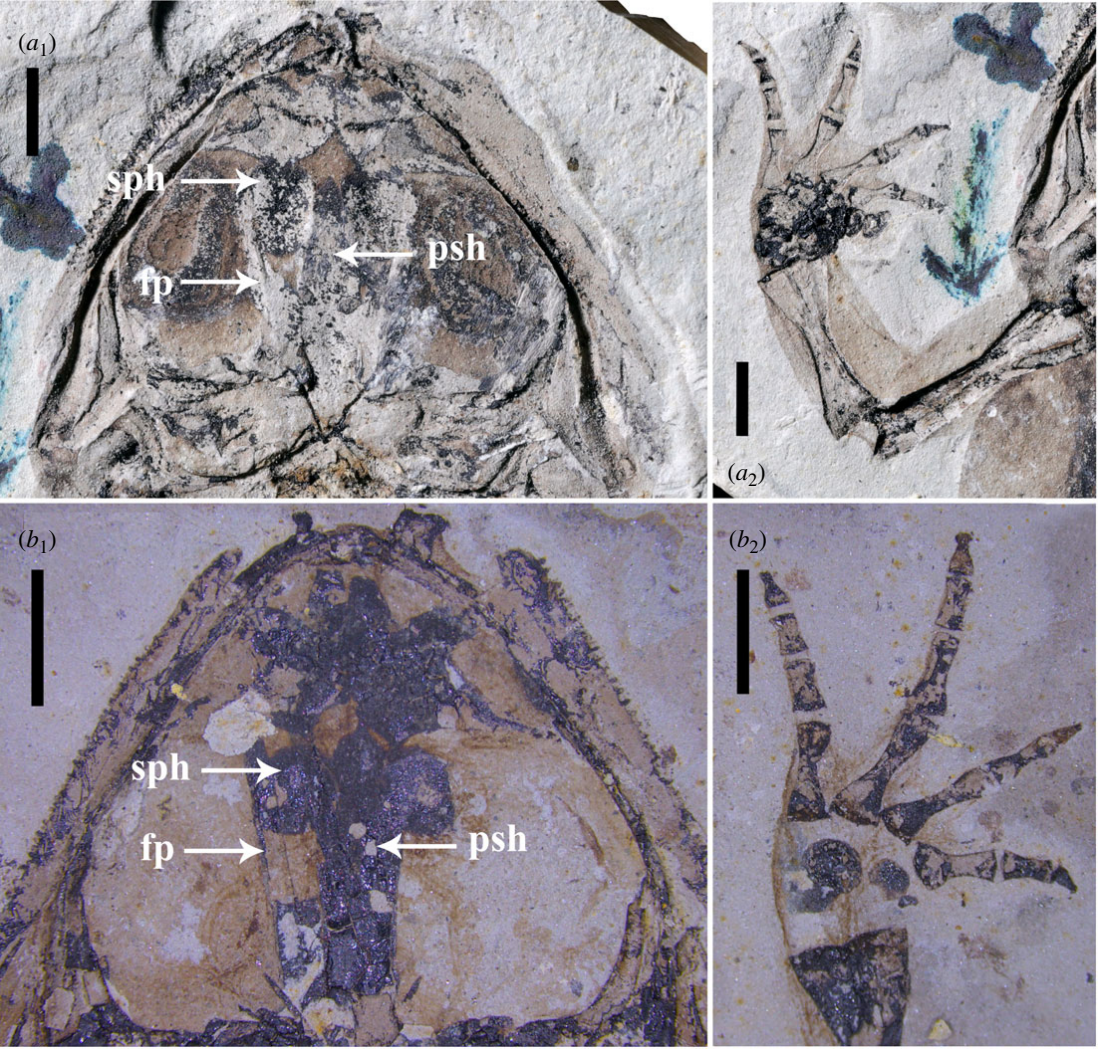
Close-ups of holotype (JQ-HX-QW-01) and paratype (JQ-HX-QW-02A) of *Gansubatrachus qilianensis* showing their different developmental stages: *a*_1_, *b*_1_, skulls (fp, frontoparietal; psh, parasphenoid; sph, sphenethmoid); *a*_2_, *b*_2_, Forelimbs (different carpal ossification states were shown in the two specimens). Scale bar: 2 mm.

The slender frontoparietals of JQ-HX-QW-02 compared to the more robust ones of the holotype (JQ-HX-QW-01) suggest that the former specimen represents an earlier developmental stage, yet because osteological differences due to sexual dimorphism also occur in anurans [35], we cannot dismiss the possibility that the holotype represents a male. Most adult anurans have a fully ossified carpus, with varying degrees of ossification at different developmental stages [11,55], although a few species of *Leiopelma* and some small or aquatic species retain at least some cartilaginous carpals as adults [47,56–59]. Carpal ossification in the holotype and paratype of *Gansubatrachus* is significantly different whereas the holotype (JQ-HX-QW-01) has the full set of carpal elements ossified (figure 5*a*_2_) [34], the paratype (JQ-HX-QW-02) shows only the distal ulnar and radial elements ossified (figure 5*b*_2_). The lesser degree of carpal ossification may suggest a younger developmental stage for the paratype, but again, sexual dimorphism as a potential source of osteological differences cannot be ruled out. After considering all these factors, disparities in ossification suggest that the skeleton of the paratype is not fully mature and indicates a younger stage compared to the holotype.

### (b) Life-history traits of a cretaceous frog

The circular structures are clustered at the right side and are band-like at the left side in the abdominal cavity of the fossil specimen (figures 2*a* and 3*a*,*b*). They are uniform in size and form a round black carbon film, resulting in dark fossil traces that are likely attributed to their high protein content [16]. Considering the typical diet of extant anurans, including insects, other small arthropods, worms and even vertebrates, it is unlikely that these structures represent preserved seeds, algae or prey within the stomach [9,60]. Among extant anurans, the number of eggs ranges from five or six in several microhylid species to about 30 000 in the true toad *Rhinella marina* [61], whereas egg size generally ranges between 1 and 10 mm and ovum size between 0.6 and 7.0 mm [62]. The size of each fossil ‘egg’ is about 0.8 mm, within the range of ovum size of living species. Therefore, by combining characteristics such as morphology, position, size and arrangement, the circular structures preserved on the paratype of *Gansubatrachus qilianensis* are likely to be ovarian follicles and eggs.

The presence of fossilized frogs with well-preserved soft tissues or eggs is exceedingly scarce in the geological record. The soft-body impression of the current specimen distinguishes it from the Spanish frog fossils, which preserved layered body soft tissue of a different nature [60]. Additionally, the carbonized eggs preserved on the specimen of *Gansubatrachus qilianensis* are also distinct in size and shape compared to the traces previously identified as air sacs [15,16]. It is worth mentioning that the present eggs are somewhat similar to the eggs reported in fossil tadpoles from the Lower Miocene of Turkey, while the eggs found in the fossil frog exhibit greater anatomical plausibility compared to those present in the abdomen of tadpoles.

In living anurans, as in all vertebrates, the female reproductive system is generally divided into two separate parts: the ovary and oviduct [63]. The ovary is typically located on the posterior side of the body and composed of ovarian follicles, and each ovarian follicle contains a developing egg. The eggs will occupy the body cavity as they mature during the reproductive period, and undergo significant distortion when passing through the oviduct towards the uterus [64]. Here, on the present paratype (JQ-HX-QW-02A) of *Gansubatrachus*, the ovarian follicles are tightly clustered together, and enclosed within the right side of the body cavity at a specific distance from the contoured skin border (figure 3*b*). While a string of eggs on the left side is clearly squeezed and deformed (figure 3*a*), it is likely that they were passing through the oviduct. However, considering the preservation of the current fossil specimen, it is possible that the lower abdomen may have undergone mutilation during burial compression or decay processes [65], potentially resulting in the failure to preserve soft tissue within the abdomen. Furthermore, female anurans typically participate in amplexus with a male partner to facilitate the release of eggs [66], so in these cases a female frog barely lays eggs without being amplexed [67]. The evenly sized eggs preserved in groups and those likely in the oviduct indicate that the specimen JQ-HX-QW-02 was a gravid female whose ovarian eggs have matured and begun to be released into the oviduct [23,68].

Although sexual maturity is achieved before full skeletal maturity in most non-avian vertebrates [69], its documentation in the fossil record is sparse [70]. Specimen JQ-HX-QW-02 clearly indicates that in *Gansubatrachus* sexual maturity was reached before the skeleton was fully developed and ossified, as evidenced by the carbonized eggs and the incomplete ossification of the carpus and the cartilaginous long bone epiphyses, which are typically interpreted as a sign of immaturity in anurans [71]. The phenomenon of indeterminate growth, characterized by the continuation of growth even after reaching sexual maturity, is widely observed among extant amphibians [72]. The discovery of the paratype of *Gansubatrachu*s *qilianensis* provides direct evidence that this phenomenon has a history extending back to approximately 115 Ma and likely ingrained in the evolutionary history of frogs.

It should be noted that cartilaginous epiphyses, but not a cartilaginous carpus, could alternatively be interpreted as the primitive adult condition for anurans, because several early anurans (or stem-anurans) from the Triassic–Early Cretaceous also lack ossified long bone epiphyses and yet have been considered mature [3,12,73–75]. This interpretation is also supported by the mostly cartilaginous epiphyses of adult salamanders [12,76,77], which are the sister group of anurans. The phylogenetic relationships of *Gansubatrachus* [34] (electronic supplementary material), as well as its antiquity, make this interpretation at least likely.

Finally, the cartilaginous epiphyses observed both in the holotype JQ-HX-QW-01 and paratype JQ-HX-QW-02 of *Gansubatrachus qilianensis* could also be related to aquatic habits, as is also suggested by the webbed manus [34] (figure 5*a,b*) and the limb proportions (electronic supplementary material), which are consistent with those of many aquatic anurans [4,78]. This is also supported by aquatic salamanders having epiphyses proportionally more cartilaginous than terrestrial species [77]. However, it should be noted that similar limb proportions are also present in jumping species and *Gansubatrachus qilianensis* do not seem to show further aquatic adaptations in its skeleton, suggesting that it was rather generalized. It should also be noted that swimming crown-group anurans, such as extant and extinct pipids, water frogs, and paradoxical frogs, are typically characterized by strongly ossified long bone epiphyses [36,50]. Cartilaginous epiphyses in sexually mature *Gansubatrachus qilianensis* are interpreted as a primitive feature that is also consistent with aquatic habits in early anurans.

### (c) Cause of death and taphonomy

The primary causes of death of extant adult frogs are environmental stress, predation, starvation during hibernation, mating behaviour and old age [79]. Most of these causes are difficult to verify in fossils due to unpreserved, informative physical or physiological features [80], whereas some of them can be inferred from the sedimentary environment and state of preservation [79,81]. Environmental factors often force organisms to flee or cause sudden mass death. However, the strata where JQ-HX-QW-02 was found, which is interpreted as being deposited under a low hydrodynamic environment regime (grey-green mudstone and horizontal stratification and development) and has yielded scarce frog fossils [34]. In the modern world, the scarcity of amphibians often serves as an indicator of environmental stressors. Factors such as the occurrence of algal blooms, abrupt fluctuations in water conditions, and exposure to toxic gases possess the potential to result in amphibian mortality, leaving discernible evidence in sedimentary deposits [79]. However, considering the inherent sensitivity of modern amphibians to environmental changes, their reproductive activities typically occur within relatively secure environments [72]. Therefore, while it is not possible to completely exclude the influence of abrupt environmental factors, it is still considered likely that the cause of death was unrelated to environmental factors.

**Figure 6.**
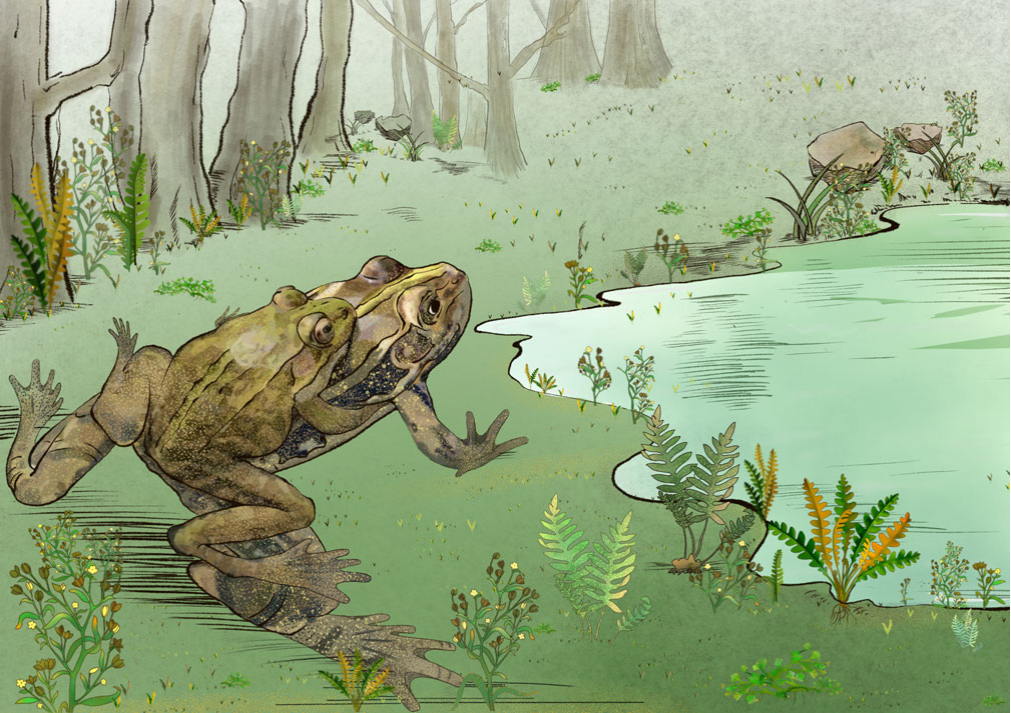
Reconstructed Early Cretaceous living environment of the *Gansubatrachus qilianensis*.

The well-preserved fossil JQ-HX-QW-02 shows neither signs of predation nor of advanced age (on the contrary, the immature skeleton suggests a relatively young age). Further, the presence of numerous eggs with some of them in the oviduct, indicates that this female was neither hibernating nor starving. The most likely cause of death for the female represented by JQ-HX-QW-02 is drowning or exhaustion in relation to mating, constituting the first Mesozoic case of death linked to mating behaviour (figure 6). This latter cause of death has also been considered to be a major cause of death in some Cenozoic anurans [79,81–85].

The condition of an organism shortly after death determines whether it is successfully preserved as a fossil, and it is how it appears to decompose when it dies in water that matters most [79]. Modern burial experiments show that soft bodies decompose under the action of bacteria even in anoxic environments [86]. But anoxic environments can reduce bioturbation and increase the chances of well-preserved fossils. Among the fossil record, few fossil vertebrates, especially anurans, are completely preserved with fully articulated skeletons, unless the sedimentary burial is under non-disturbance conditions. The preservation conditions of soft body tissues are more stringent, usually associated with their early interaction with sediments or bacterial mats during the decay process [87]. Additionally, deep water and low temperature environments can impede re-floating of the decaying carcasses, thereby reducing the likelihood of limb fragmentation [79,81]. Fish burial experiments suggest that most carcasses tend to remain at the bottom without resurfacing at water temperatures below approximately 16°C [88]. The reconstructed average annual temperature during the Aptian–Albian period in the Jiuquan Basin was approximately 15°C [89], potentially with lower water temperatures, which may contribute to the favourable preservation of the current fossil specimens.

Moreover, the combination of grey-green mudstone and horizontal bedding is often associated with relatively deep and tranquil depositional environments. The preservation of specimen JQ-HX-QW-02, characterized by intact skeleton and slight pelvic displacement, can be attributed to the weak hydrodynamic conditions that followed sediment adhesion and the influence of lower lake temperatures. And the exceptionally preservation of soft body parts and carbonized eggs *in situ* appears to be related to the presence of alumino-silicon in the surrounding depositional environments [90,91].

## Data Availability

The data are provided in electronic supplementary material [92].
