# Correction to: ‘A cretaceous frog with eggs from northwestern China provides fossil evidence for sexual maturity preceding skeletal maturity in anurans’ (2024), by Du *et al.*

**DOI:** 10.1098/rspb.2024.0528

**Published:** 2024-03-25

**Authors:** Baoxia Du, Jing Zhang, Raúl Orencio Gómez, Liping Dong, Mingzhen Zhang, Xiangtong Lei, Aijing Li, Shuang Dai

**Keywords:** Anura, Early Cretaceous, northwest China, *Gansubatrachus*, sexual maturation

*Proc. R. Soc. B* **291**, 20232320 (Published online 7 February 2024). (https://doi.org/10.1098/rspb.2023.2320)

Thank you for bringing to our attention the violation of the basic rules of the International Code of Zoological Nomenclature (ICZN) regarding the designation of fossil paratype in the original article. The specimen described in the paper could be considered as a topotype, since it comes from the same locality as the holotype. A correction is warranted, changing the designation from a paratype to a topotype. The term ‘paratype’ should be replaced, but also the phrase ‘we assign it as a paratype’ must be deleted to remove this unnecessary ‘assignation’. This specimen simply is a topotype and doesn't require any assignation/designation, in contrast to the type series, which must be designated. So, the term ‘paratype’ was replaced with ‘topotype’ in the article, and the phrase ‘we assign it as a paratype’ was deleted from the abstract.

